# Use of Intravitreal Bevacizumab Injection among Patients Undergoing Surgical Retinal Interventions at Tertiary Eye Hospital: A Descriptive Cross-sectional Study

**DOI:** 10.31729/jnma.5515

**Published:** 2021-09-30

**Authors:** Arjun Shrestha, Rinkal Suwal, Rajan Shrestha, Barsha Suwal, Deepak Khadka

**Affiliations:** 1 Department of Ophthalmology, BP Eye Foundation, Hospital for Children, Eye, ENT & Rehabilitation Services, Bagmati Province, Bhaktapur, Nepal; 2 Academic and Research Department, BP Eye Foundation, Hospital for Children, Eye, ENT & Rehabilitation, Bagmati Province, Bhaktapur, Nepal

**Keywords:** *bevacizumab*, *intravitreal injection*, *visual disorders*

## Abstract

**Introduction:**

Intravitreal Bevacizumab injection has now become a routine procedure for retina specialists throughout the world. Easy availability of this monoclonal antibody molecule even in Nepal has brought a revolution in the management of various retinal diseases. This study aims to find out the prevalence of the use of intravitreal Bevacizumab for retinal diseases at the tertiary eye hospital.

**Methods:**

This descriptive cross-sectional study was carried out in the retina department at a tertiary care hospital from January 2017 to December 2019 after obtaining ethical clearance from Nepal Health Research Council (Ref: 125/2020P). The sample size was calculated and the study enrolled all patients who received intravitreal Bevacizumab for retinal diseases using convenience sampling technique. Data were analyzed using Statistical Package for Social Science Version 21. Point estimate at 95% Confidence Interval was calculated, along with frequency and percentage for binary data.

**Results:**

Out of 959 total surgical retinal interventions done 296 (30.86%) at 95% Confidence Interval (27.93-33.78) patients received intravitreal Bevacizumab. Out of total intravitreal Bevacizumab injections, 143 (36.7%) injections were given to retinal vein occlusions patients, 127 (32.6%) injections were given to diabetic retinopathy patients and 66 (17%) injections was given to age-related macular degeneration patients. Males 176 (59.5%) outnumbered the females 120 (40.5%) in receiving intravitreal Bevacizumab. Mean baseline Logarithm of the Minimal Angle of Resolution visual acuity, 1.1, improved to, 0.75, after 3 months of intravitreal Bevacizumab.

**Conclusions:**

Intravitreal Bevacizumab was one of the commonest retinal interventions used. Retinal vein occlusion, diabetic retinopathy, and age-related macular degeneration were the commonest retinal diseases needing intravitreal Bevacizumab.

## INTRODUCTION

Intravitreal Bevacizumab (IVB) is widely used for the treatment of retinal diseases in developing countries, because of its easy availability and affordability.^1^ Studies have revealed both efficacy and safety of Bevacizumab in macular edema due to Diabetic retinopathy (DR), Age-related macular degeneration (AMD), and retinal vein occlusion (RVO).^2-5^ Multiple doses are often needed for retinal pathologies. Therefore, economic burden and choice of agent are still admissible questions. This situation inflicts a colossal burden on healthcare systems, especially in countries having lower per capita income.^6^ Although IVB is commonly performed procedures in tertiary eye care facilities also in Nepal, the profile of patients and the distribution of retinal diseases needing IVB are not clearly investigated.

This study aims to find out the prevalence of the use of intravitreal Bevacizumab profile for retinal diseases at the tertiary eye hospital.

## METHODS

This was a descriptive cross-sectional study conducted at the Hospital for Children, Eye, Ear, ENT, and Rehabilitation Services (CHEERS) in Bhaktapur reviewing all the medical records who received IVB from January 2017 to December 2019. Nepal Health Research Council (NHRC) approved the ethical clearance (Reference No: 125/2020 P) to conduct the study, and we followed the tenets of the Helsinki declaration. Informed consent was waived off in this study as it was a retrospective study, and hospital records were reviewed. We included the patients who received IVB for retinal problems only. Patients receiving combined intravitreal Corticosteroid and Bevacizumab for retinal problems and intravitreal Bevacizumab for non-retinal diseases were excluded in the study.

The sample size was calculated using the formula,

n = Z^2^ × p × q / e^2^

  = (1.96)^2^ × {0.5 × (1-0.5)} / 0.05^2^

  = 0.9604/0.0025

  = 385

Where,

n = required sample size
Z = 1.96 at 95% Confidence Interval (CI)
p = prevalence 50%,
q = 1-p
e = margin of error, 5%

Adding a 10% none response rate, the total sample size was found to be 424. Doubling the sample size, we get 848. But 959 samples were taken.

We used convenience sampling and included all patients who received IVB for retinal diseases. An especially designed checklist was filled after retrieving data from the outpatient card. Snellen's visual acuity was converted to log MAR visual acuity to make it more standardized for statistical computing. Post injection visual status was assessed after 1 month and 3 months of IVB as in the study protocol adopted in Thailand.^7^

Data were entered into Microsoft Excel and the Statistical Package for the Social Sciences version 21 to study the different variables and statistical tests. The variables studied were the demographic characteristics of retinal patients, the distribution of retinal diseases needing IVB, and their visual outcome. Demographic characteristics and distribution of retinal diseases were illustrated in terms of frequencies, means, percentages, and standard deviations (SD) or range.

## RESULTS

Out of 959 total surgical retinal interventions done 296 (30.86%) at 95% CI (27.93-33.78) patients received intravitreal Bevacizumab.

The total number of IVB was 680 and the total number of retinal patients was 296. Eighty-seven (29.19%) patients received IVB in both eyes. Males outnumbered females in IVB utilization by the ratio of 3:2. The mean number of IVB was 2.1±1.5 per eye (Table 1).

**Table 1:** General characteristics of patients (n = 296).

Demography	n (%)
Male	176 (59.5)
Female	120 (40.5)
Number of patients who had both eyes injected	87 (29.19)
Number of patients who had single eye injected	211 (70.80)

RVO is the commonest retinal problem needing IVB; followed by DR and AMD (Table 2).

**Table 2:** Age pattern and distribution of retinal diseases for which intravitreal Bevacizumab was used.

Indication	n (%)	Age (Mean/SD) in Years
AMD	66 (17)	72.04 (8.62)
RVO	143 (36.7)	63.58 (12.22)
DR	127 (32.6)	59.12 (9.53)
Others	49 (12.6)	55.26 (17.92)

Mean visual acuity (VA) improved from baseline Log MAR 1.1 to 0.75 after 3 months of injection (Table 3).

**Table 3:** Visual acuity according to indication.

Indication	Mean	Mean VA at 3 months	Mean difference
All	1.1	0.75	0.26
BRVO	1.17	0.65	0.52
CRVO	1.36	0.76	0.59
AMD	1.55	1.33	0.22
DR	1.07	0.74	0.32
Others	1.28	0.33	0.95

## DISCUSSION

To our best knowledge, this study is the first of its kind showing detailed data of intravitreal Bevacizumab at a tertiary level eye hospital in Nepal. Intravitreal Bevacizumab injections are increasingly used in various retinal pathologies in Nepal.

IVB was proven to be as safe and effective in various retinal diseases by various major studies. IVB is relatively affordable and it reduces the financial burden of multiple injections which is very crucial in developing countries with limited per capital expenditure capacity.^8^

RVO, DR, and AMD were the commonest retinal diseases needing IVB in our study. A retrospective study from another hospital in Nepal also showed similar retinal diseases patterns needing IVB.^8^ With increasing urbanization, change in diet and lifestyle, the prevalence of hypertension and diabetes is also increasing in Nepal. The rising trend of hypertension and diabetes in our community could have led to vision-threatening retinal diseases like RVO and DR in Nepal. In contrast, DR and AMD needed IVB more compared to RVO in India and Oman.^8,10^ It could be due to different comorbidity patterns of diabetes and hypertension in India and Oman compared to Nepal. There is an improvement in the life expectancy of Nepalese people than in previous years, and hence it could be a potential risk factor of AMD. AMD with morphological findings of choroidal neovascularization might have been underdetected in our setup. The possible reason may be that poor vision in the elderly is considered relatively normal. Due to transport difficulty and little negligence in health care; it might have resulted in a bit of a lower number of AMD in our study.

There were more males compared to females receiving IVB at our hospital. This similar gender disparity was also observed in other studies in Nepal.^5,9^ Nepal is a male dominant country, and probably they tend to seek medical attention earlier than females.^11^

The mean age of patients needing IVB varied according to the type of retinal problems. AMD was found more in the advanced age group compared to RVO and DR in Nepal. This finding highlights the role of aging in the prevalence of AMD. Thirty percent of patients received IVB in both eyes. This bilateralism disease severity is mainly consequent of DR and AMD.

Mean baseline visual acuity was 1.1, which improved to mean post-injection visual acuity of 0.87, 0.75, respectively after 1 and 3 months of injections. We couldn't find many patients continuing IVB for a longer duration. The mean number of injections in our hospital was 2.1 ±1.5. This finding is similar to a study done in India, where the mean number of injections was 2.16±1.56.^7^ The number of injections is fewer when we compare with the real-world data of patients from the developed world. This could be due to declining motivation to receive treatment after three consecutive doses, poor financial background of our patients, poor access to services and hospital facilities, and limited health insurance in Nepal. The necessity for multiple injections to maintain stable vision is a major challenge in our setup due to cost factors. Per dose of IVB is about 60 USD and a loading dose of 3 consecutive injections will be very high compared to the unit cost of cataract surgery in Nepal. Poorly controlled glycemic status in DR and different pathogenesis in AMD might have led to poor vision gain in these diseases. This finding was similar to the study done in Thailand.^7^

Our study has many shortcomings as expected in any retrospective study. Follow up period was limited to 3 months only as there was a substantial number of patients drop out after 3 months.

Other limitations of our study included limited sample size, single-institution study, and convenience sampling method used.

## CONCLUSIONS

Intravitreal Bevacizumab was one of the commonest retinal interventions used. Retinal vein occlusion, diabetic retinopathy, and age-related macular degeneration were the commonest retinal diseases needing intravitreal Bevacizumab in our study. For chronic diseases such as DR and AMD, that require frequent dosing, Bevacizumab may still be expensive in the Nepalese context. We recommend a primary and secondary level of preventive approach for risk factors for RVO and DR like hypertension, diabetes, and obesity in the community.
